# Hepatotoxicity prevention in Acetaminophen-induced HepG2 cells by red betel (*Piper crocatum* Ruiz and Pav) extract from Indonesia via antioxidant, anti-inflammatory, and anti-necrotic

**DOI:** 10.1016/j.heliyon.2020.e05620

**Published:** 2021-01-04

**Authors:** Chrismis Novalinda Ginting, I Nyoman Ehrich Lister, Ermi Girsang, Wahyu Widowati, Dewani Tediana Yusepany, Alya Mardhotillah Azizah, Hanna Sari Widya Kusuma

**Affiliations:** a Faculty of Medicine, Universitas Prima Indonesia, Jl. Belanga No. 1, Medan 20118, North Sumatera, Indonesia; b Faculty of Medicine, Maranatha Christian University, Jl. Prof. Drg. Surya Sumantri No. 65, Bandung 40163, West Java, Indonesia; c Aretha Medika Utama, Biomolecular Research Center, Jl. Babakan Jeruk II No.9, Bandung 40163, West Java, Indonesia

**Keywords:** Red betel leaves extract, Acetaminophen, HepG2 cells, Hepatoprotective, Biochemistry, Immunology, Inflammation, Natural product, Pharmaceutical science

## Abstract

Acetaminophen (APAP) is a widely used analgesic, but it may cause liver injury (*hepatotoxicity)* via oxidative stress that induced by *N*-acetyl-*p*-benzoquinone imine (NAPQI) in long term usage or overdose. Multiple inflammatory mediators were also found to contribute for this effect. Many medicinal plants was known for its antioxidant and anti-inflammatory activities and one of them is Red betel (*Piper crocatum* Ruiz and Pav) from Indonesia. In this study, the red betel leaves extract (RBLE) protective effect against APAP-induced HepG2 cells was determined. APAP-induced HepG2 as hepatotoxicity cell model was treated with RBLE at 25 and 100 μg/mL. Protective effects of RBLE toward hepatotoxicity were evaluated by several parameters: tumor necrosis factor-α (TNF-α) concentration, reactive oxygen species (ROS) level, live cells percentage, apoptotic cells percentage, necrotic cells percentage, death cells percentage, CYP2E1 and GPX gene expression. The RBLE treatments (both 25 and 100 μg/mL) increased CYP2E1 and GPX gene expression also live cells percentage, while decreased ROS level, TNF-α concentration, also the percentage of death and necrotic cells. Red Betel leaves ethanol extract has hepatoprotective effect via anti-inflammatory, anti-necrotic, and antioxidant potency in liver injury model.

## 1 Introduction

For many problem of drugs use, liver injury was continues to be a problem. It was represents a major challenge in designing potential therapies (Noh et al., 2015). Acetaminophen (paracetamol, APAP) is considered as first line analgesics. However, excessive use of APAP leads to liver injury even liver failure in human (Ganey et al., 2007; Ni et al., 2012). In small percentage, the cytochrome P450 2E1 (CYP2E1) enzymes was oxidizing the APAP and form N-acetyl-p-benzoquinone imine (NAPQI), a highly reactive intermediate, which is detoxified by covalent binding with glutathione (GSH). However, in APAP poisoning, it will generates excess NAPQI which evokes the GSH depletion that binds to macromolecules triggering oxidative stress, mitochondrial dysfunction, and ultimately resulting in hepatocellular death (Salminen et al., 2012; Uzi et al., 2013; Yuan et al., 2016). Although the mechanisms underlying hepatotoxicity that induced by APAP still unclear, some evidences was indicate that inflammation mediators such as tumor necrosis factor-α (TNF-α) also oxidative stress was contribute to the APAP-induced acute liver damage pathology process (Uzkeser et al., 2012; Dragomir et al., 2012).

One of betel in Indonesia namely red betel (*Piper crocatum* Ruiz and Pav) has medicinal function and used as medicine since its introduce as medicinal plants producer in Blunyahrejo (Rinanda and Alga, 2012). It can be used to treat diabetes, gout, hepatitis, hypertension, and eye inflammation (Anugrahwati et al., 2016). In previous study, red betel leaves were found to have some secondary metabolite content like flavonoids, alkaloids, tannins, saponins, triterpenoids steroids, quinones, polyphenolics, and essential oil groups (Arambewela et al., 2005; Wulandari et al., 2018). In addition, red betel contains phenolic compounds in the form of hydrochavicol, cavibetol acetate and eugenol (Swapna et al., 2012; Dervis et al., 2017). In previous studies, red betel leaves extract (RBLE) was shown have anti-inflammatory properties (Misra et al., 2009); antioxidant activity (Lister et al., 2019a); and also have anticancer activity especially cervical cancer (Widowati et al., 2013) and breast cancer (Zulharini et al., 2018).

In this study, RBLE potential to suppress liver injury in APAP-induced HepG2 cells was conducted. The parameters that observed in this study was Reactive Oxygen Spesies (ROS) level; TNF-α level; Cytochrome P450 Family 2 Subfamily E Member 1 (CYP2E1) and Glutathone Peroxidase (GPX) gene expression; apoptotic, necrotic cells, and death cells percentage.

## 2 Materials and methods

### 2.1 Preparation of red betel leaves extract

The red betel (*P. crocatum* Ruiz and Pav) leaves that used in this study was obtained from Pabuaran Cilendek Timur, Indonesia and has been identified by Herbarium Bogoriense, Botanical Field Research Center for Biology-Indonesian Institute of Science, Indonesia. RBLE preparation was done by using maceration method. RBLE was obtained from our previous research and stored at -20 °C (Lister et al., 2019a, 2019b).

### 2.2 HepG2 cells culture and APAP-Induced HepG2

The cells that used in this study is human hepatocellular carcinoma (HepG2) cell line (ATCC, HB-8065™) from Aretha Medika Utama Biomolecular and Biomedical Research Center, Bandung, Indonesia. It was grown in complete medium with composition: Modified Eagle Medium (MEM) (Biowest, L0416-500), fetal bovine serum (FBS) (Biowest, S1810) as much as 10% (v/v), antibiotic-antimycotic (Gibco, 15240062) as much as 1% (v/v), also nanomycopulitine (Biowest, LX16) addition) as much as 1% of (v/v). Acetaminophen (Sigma Aldrich, A7085) with concentration at 40 mM was used to induce the hepatotoxicity. When the cells were confluent, it was rinsed using PBS and detached using trypsin-EDTA (Gibco, 25200072) with incubation at 37 °C. In 6 well plates, the cells was seeded (5 × 10^5^ cells/well) and then incubated at the same temperature with 5% of CO_2_ for 24 h. The cells was induced by RBLE and incubated again for 24 h. According to the treatment, it was divided into 5 groups: I) Normal Cells; II) DMSO1%; III) APAP 40 mM; IV) APAP 40 mM + RBLE 25 μg/mL; V) APAP 40 Mm + RBLE 100 μg/mL. Then it was centrifuged at 1600 rpm for 10 min. The supernatant was collected as sample for the Elisa assay (Luo et al., 2016; Aouache et al., 2018; Lister et al., 2019b).

### 2.3 Total protein assay

Bovine Serum Albumin (BSA) (Sigma Aldrich, A9576) was used as standard in this method. Briefly standard solutions as much as 20 μL also same volume for the samples was mixed with Quick Start Dye Reagen 1X (Biorad, 5000205) as much as 200 μL into each well in 96 well plate. The mixture then incubated at room temperature for around 5 min. The wavelength at 595 nm was used to determine the mixture absorbance by using microplate reader (Multiskan™ GO Micro plate Spectrophotometer, Thermo Scientific, 51119300) at 595 nm. The result from this assay was used for normalization of TNF-α data calculation (Pluemsamran et al., 2012; Widowati et al., 2019a).

### 2.4 TNF-α assay

This assay was measured using ELISA assay (BioLegend, 421701) and done according to the manufacturer's kit manual. Based on the manual, wavelength at 450 nm was used to determine the absorbance using microplate reader (Widowati et al., 2019a).

### 2.5 Apoptotic activity assay

The assay was conducted using methods that reported by Widowati et al. (2019b). Treated and control HepG2 cells were washed using PBS 1x and harvested using trypsin-EDTA for apoptotic assay. The pellet was washed using Annexin Binding Buffer 1X (Miltenyi Biotec, 130-092-820) 500 μL and stained with Annexin V-FITC (BioLegend, 79998) and Propidium Iodide (BioLegend, 79997). Cells were incubated at 37 °C for 30 min in the dark. Cells were later suspended in Annexin Binding Buffer 1x. The HepG2 cells apoptotic percentage were analyzed using MACSquant Analyzer 10 (Miltenyi Biotec).

### 2.6 Reactive oxygen species (ROS) assay

HepG2 cells were digested with trypsin-EDTA after cultured around 7 days and 2.5 × 10^4^ cells/0.5 mL were incubated for 45 min in 20 μM DCF-DA at 37 °C and incubated again for 4 h in RBLE. Based on Prahastuti et al. (2019) and Girsang et al. (2019), the 2′,7′–dichlorofluorescin diacetate (DCFDA)–Cellular Reactive Oxygen Species Detection Assay Kit (Abcam, ab113851) was used to measured intracellular ROS with modifications.

### 2.7 The expression of GPX and CYP2E1 gene assay

Cells that has been harvested was processed for RNA isolation that will be used for futher assay. It was done by using the Aurum™ Total RNA mini Kit (Bio-Rad, 732-6820). RT-qPCR (Clever, GTC96S) was used to analyze the gene expression include the β-actin gene that constitutively expressed (Afifah et al., 2019; Widowati et al., 2019b). Table 1 was shown the primer sequence and Table 2 was shown RNA concentration and purity.

**Table 1:** RT-PCR details of β-Actin, CYP2E1, and GPX gene.

Gene Symbols	Primer Sequences (5′ to 3′) Upper strand: Sense Lower strand: Antisense	Annealing (°C)	Cycle	References
β-Actin	5′-TCTGGCACCACACCTTCTACAATG-3′ 5′-AGCACAGCCTGGATAGCAACG-3′	63	40	Widowati et al. (2019b) Afifah et al. (2019)
CYP2E1	5′-GTTCTTTGCGGGGACAGAGA-3′ 5′-GAGGGTGATGAACCGCTGAA-3′	59	40	Kim et al., 2003
GPX	5′-CCAAGCTCATCACCTGGTCT-3′ 5′-TCGATGTCAATGGTCTGGAA-3′	59	40	Ugusman et al. (2011)

**Table 2:** RNA concentration and purity.

No.	Sample	Concentration (ng/μL)	Purity (Absorbance 260/280)
1.	Control cells	92.90	2.3212
2.	Positive control	90.10	2.0904
3.	Positive control + RBLE 25 μg/mL	36.20	1.9676
4.	Positive control + RBLE 100 μg/mL	40.00	2.0366

### 2.8 Statistical analysis

All data were obtained after doing it in triplicate. When the data has normal distribution, it was analyzed using ANOVA and *Post Hoc* Test using Tukey HSD with p < 0.05 while data didn't has normally distributed were analyzed with Kruskal Wallis and *Post Hoc* Test Mann Whitney using SPSS software (version 20.0). The data were presented as mean ± standard deviation.

## 3 Result

### 3.1 RBLE effect towards TNF-α concentration in APAP-induced HepG2 cells

**Figure 1:**
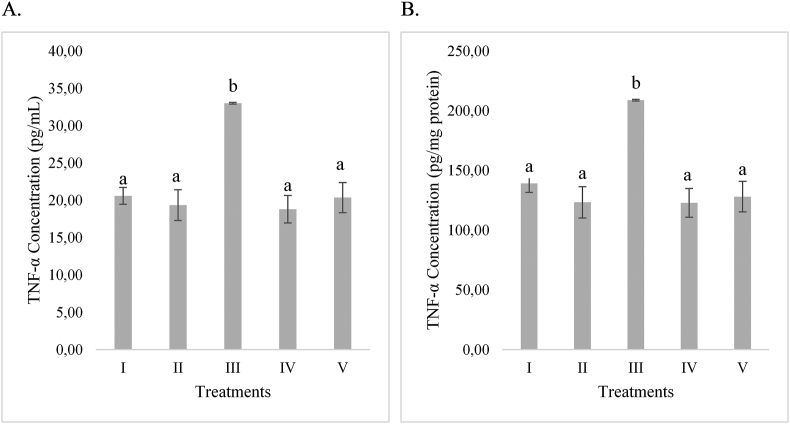
RBLE treatment effect toward TNF-α concentration in APAP-induced HepG2 cells as hepatotoxicity model. (A) TNF-α concentration (pg/mL) on hepatotoxicity model. (B) TNF-α concentration (pg/mg protein) on hepatotoxicity model. ∗Data was included as mean ± standard deviation. I) Normal cells as negative control; II) Normal cells + DMSO 1% as vehicle control; III) APAP-induced cells (Positive control); IV) Positive control + RBLE 25 μg/mL; V) Positive control + RBLE 100 μg/mL. Significance among treatments toward TNF-α concentration was presented as different letters (a,b) based on Tukey HSD post hoc test (P < 0.05).

APAP was increased the TNF-α concentration in HepG2 cells. When RBLE treatment was added, it was found can decrease the TNF-α concentration (Figure 1). Based on the result, RBLE has potential to supress the TNF-α production in HepG2 cells that induced by APAP.

### 3.2 Effect of RBLE towards apoptotic, necrotic, and cell death in APAP-induced HepG2 cells

**Figure 2:**
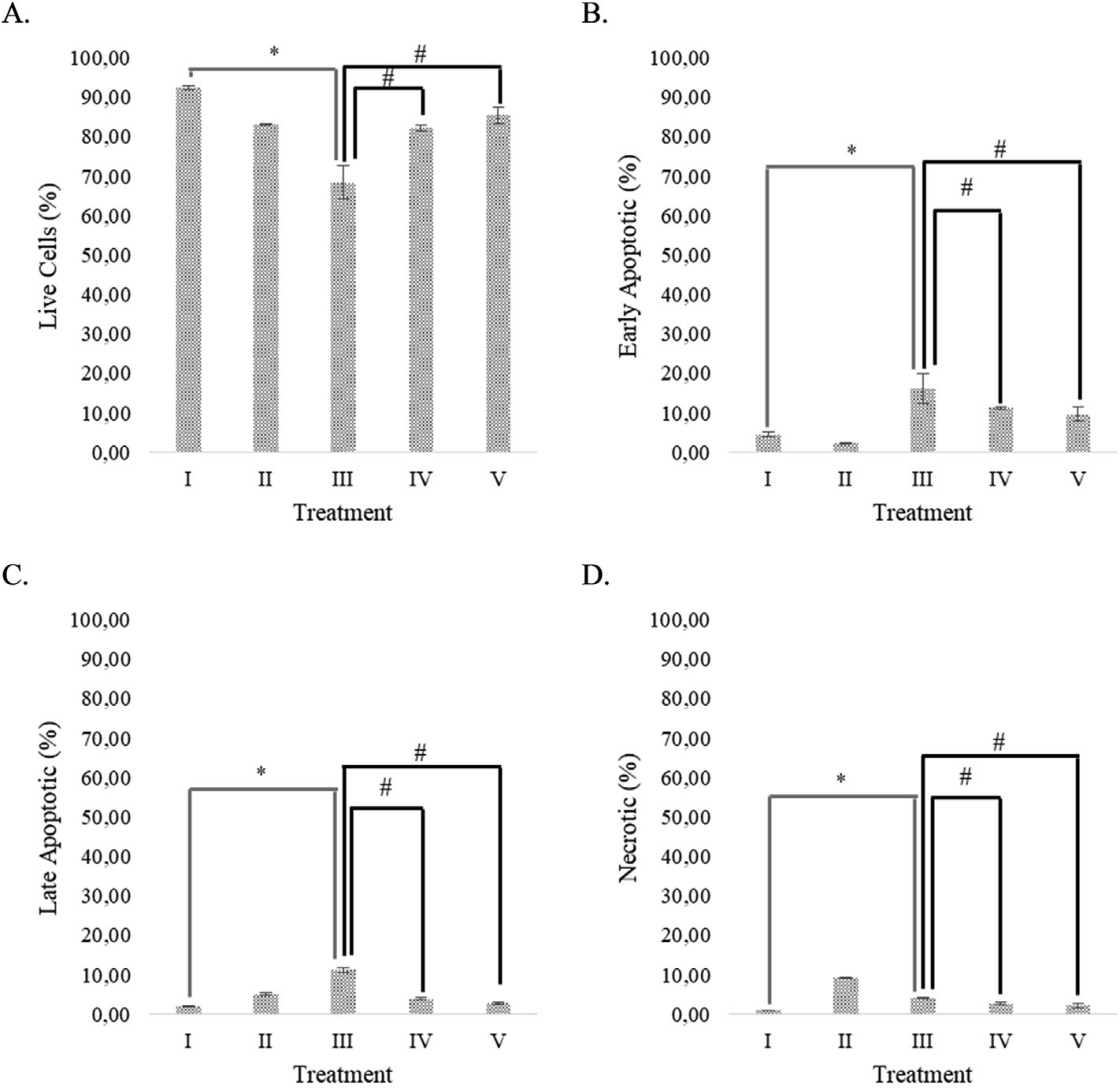
RBLE effect toward apoptotic, necrotic, dead cells in hepatotoxicity model. (A) Live cells on hepatotoxicity model. (B) Early apoptotic on hepatotoxicity model. (C) Late apoptotic on hepatotoxicity model. (D) Necrotic on hepatotoxicity model. ∗Data was included as mean ± standard deviation. I) Normal cells as negative control; II) Normal cells + DMSO 1% as vehicle control; III) APAP-induced cells (Positive control); IV) Positive control + RBLE 25 μg/mL; V) Positive control + RBLE 100 μg/mL. There are significant different between all groups based on Kruskal-Wallis Test (P < 0.05) and Mann-Whitney Test (P < 0.05). It was marked as single star (∗) marks for the statistical difference between positive control and negative control while the hashtag (#) mark for statistical difference between treatment and positive control.

APAP decreased live cell percentage compare to normal HepG2 cells (Figure 2A). RBLE treatment decreased the percentage of apoptotic and necrotic significantly in APAP-induced HepG2 cells (Figure 2B–D). RBLE treatment can increase the live cells percentage also reduce the percentage of necrotic and dead cells in HepG2 cells that induced by APAP.

### 3.3 RBLE effect towards ROS level in liver injury model

**Figure 3:**
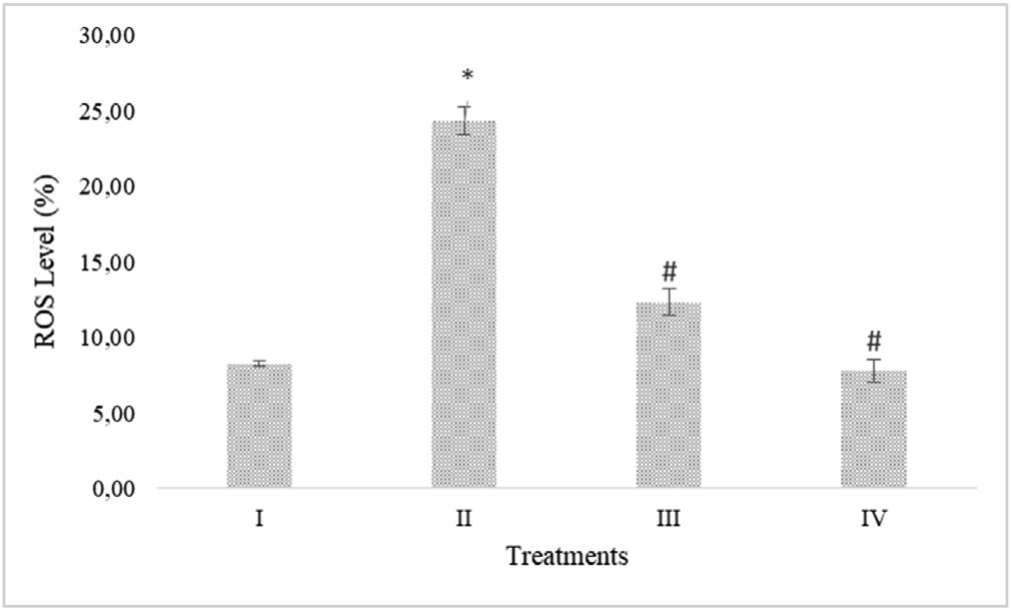
RBLE effect toward ROS level in hepatotoxicity model. ∗Data was included as mean ± standard deviation. I) Normal cells (Negative control); II) Normal cells + DMSO 1%; III) APAP-induced cells (Positive control); IV) Positive control + RBLE 25 μg/mL; V) Positive control + RBLE 100 μg/mL. Based on Kruskal-Wallis Test (P < 0.05), there are significant different among groups. It was marked as single star (∗) for statistical difference between positive control and negative control also hashtag (#) for statistical difference between treatment and positive control.

ROS level increased significantly after APAP induction and reduced significantly when injured HepG2 cells were treated with RBLE (Figure 3). RBLE had potential to decrease ROS level in liver injury model.

### 3.4 RBLE effect on CYP2E1 and GPX gene expression in liver injury model

**Figure 4:**
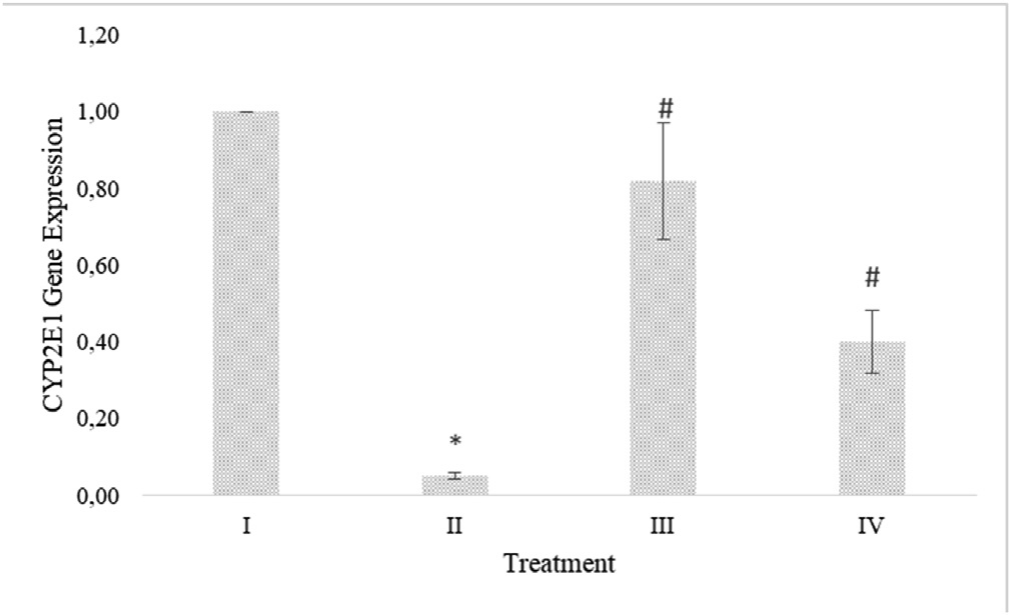
RBLE effect toward the expression of CYP2E1 gene in hepatotoxicity model. ∗Data was included as mean ± standard deviation. I) Normal cells as negative control; II) Normal cells + DMSO 1%; III) APAP-induced cells (Positive control); IV) Positive control + RBLE 25 μg/mL; V) Positive control + RBLE 100 μg/mL. Based on ANOVA (P < 0.05) and Games-Howell (P < 0.05), there are significant different among all groups. It was marked as single star (∗) marks for the difference between positive control and negative control also hashtag (#) mark for the difference between treatment and positive control.

**Figure 5:**
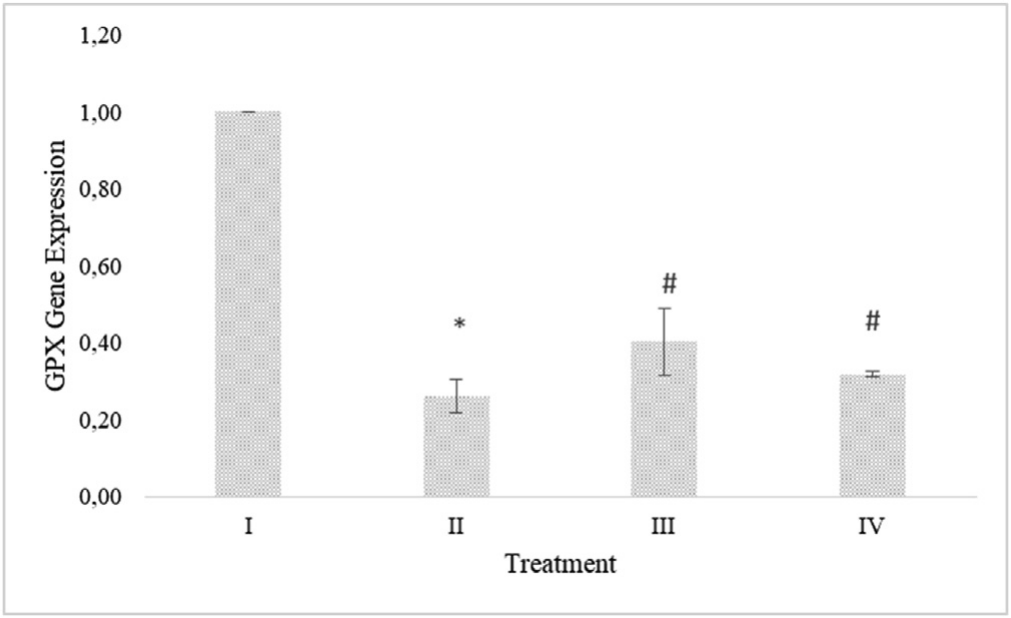
RBLE effect toward the expression of GPX gene in hepatotoxicity model. ∗Data was included as mean ± standard deviation. I) Normal cells (Negative control); II) Normal cells + DMSO 1%; III) APAP-induced cells (Positive control); IV) Positive control + RBLE 25 μg/mL; V) Positive control + RBLE 100 μg/mL. Based on Kruskal-Wallis Test (P < 0.05) and Mann-Whitney Test (P < 0.05), there are significant different between group. It was marked as single star (∗) marks as difference between positive control and negative control while hashtag (#) mark as difference between treatment and positive control.

CYP2E1 gene expression decreased significantly in APAP-induced HepG2 cells. RBLE treatments increased the CYP2E1 gene expression significantly compare to the APAP-induced HepG2 cells group (Figure 4). GPX gene expression decreased in APAP-induced HepG2 cells. RBLE treatments could increase the GPX gene expression significantly (Figure 5). RBLE treatments had ability to increase the CYP2E1 and GPX gene expression.

## 4 Discussions

Betel leaves had been known to contain many active compounds, mainly hydrochavicol, cavibetol acetate and eugenol (Begam et al., 2018). Based on previous study, it had been demonstrated that red betel leaves extract, along with its active constituents: eugenol and hydroxychavicol, can scavenging H_2_O_2_ and DPPH also reducing FRAP and ABTS radicals that indicated their antioxidant activity (Lister et al., 2019a). Eugenol also had been reported could decrease the ALT and AST activities and LDH level in liver injury model that induced by APAP (Lister et al., 2019b).

The presence of APAP toxic metabolite NAPQI caused Kupffer cells activation that leads to TNF-α release (Legert et al., 2015). TNF-α, one of inflammatory cytokine, involved in oxidative stress injury (Barman et al., 2016; Jaeschke et al., 2012). It was mediated death receptor pathway apoptosis by activating caspase 3 that act as a central effector to cleave various cellular substrates and trigger cell apoptosis eventually (Nagase et al., 2002; Truong et al., 2016). While apoptosis and necrosis frequently coexist in liver pathological conditions and the cell death balance may be dictated by the particular insult (Antoine et al., 2010).

RBLE treatment was found can decrease the TNF-α level in liver injury model based on the study result. One of active compound in RBLE, eugenol, had been studied have effect on reduction of inflammatory cells infiltration and generation of cytokines from Kupffer cells include ability to suppress TNF-α level in liver injury model (Yogalakshmi et al., 2010). Phenolic compound had anti-inflammatory effect as another study from Yuan et al. (2016) also stated that a phenolic compound ferulic acid could decrease the TNF-α level in mice induced with APAP.

Figure 2 shows that the APAP induction increased the apoptotic, necrotic, and death cells percentage, while RBLE treatments had successfully reduce death cells and maintain live cells at higher level. This data was in line with previous research that less apoptotic cells were seen in ferulic acid treatment in injury liver model (Yuan et al., 2016).

In APAP-induced hepatotoxicity model, oxidative stress played an important role and it was characterized by ROS accumulation (Nagi et al., 2010; Du et al., 2016). NAPQI, a reactive metabolite formed from APAP, could react rapidly with GSH and aggravating oxidative stress in conjuction with mitochondrial dysfunction that induced hepatocellular damage (Smith et al., 2016; Kang et al., 2017). The enzymatic antioxidant defense system known can detoxified ROS. Previous study exhibited that RBLE had antioxidant potential (Lister et al., 2019a). Based on the result, RBLE proved to suppress the ROS level in liver injury model, this result was in line with Parikh et al. (2015) that found phenolic compounds in *Brassica juncea* hydromethanolic extract such as quercetin and cathecin could reduce the ROS level in APAP-induced HepG2 cells. Thus, the RBLE hepatoprotective mechanism might result from diminishing generation of ROS.

In the metabolism of endogenous and exogenous compounds wide variety, CYP2E1 has important functions that relevant to chemical toxicity and carcinogenesis in liver (Gonzalez, 2007). ROS was one of compounds that generated by CYP2E1 that increase mitochondrial membrane permeability and lipid peroxidation, which induce apoptosis via pro-apoptotic factors release and activate caspase 3 (Lee and Wei, 2007). Based on the result, RBLE treatments decreased CYP2E1 gene expression in APAP-induced HepG2 cells, probably by its high phenolic compounds. This result was in line with previous research that in APAP-induced hepatotoxicity, the ferulic acid could inhibit the up-regulation of CYP2E1 expression (Yuan et al., 2016).

The enzymatic antioxidant defense system primary part against oxidative stress is GPX that directly eliminating ROS (Truong et al., 2016). When free radicals formed rapidly, GPX functions will become inefficient and leads to hepatocytes damage (Roh et al., 2018). GPX level can be used as indicator of the oxidative stress response (Wang et al., 2016). Based on the result, it was shown that APAP could decrease the GPX expression, however RBLE treatments could counter this effect. It was indicated that RBLE can protects cells/livers from APAP-inducer through an antioxidant defense system enhancement. Truong et al. (2016) also stated that a phenolic compound, mainly quercitrin could restore GPX expression and attenuates APAP-induced liver damage.

**Figure 6:**
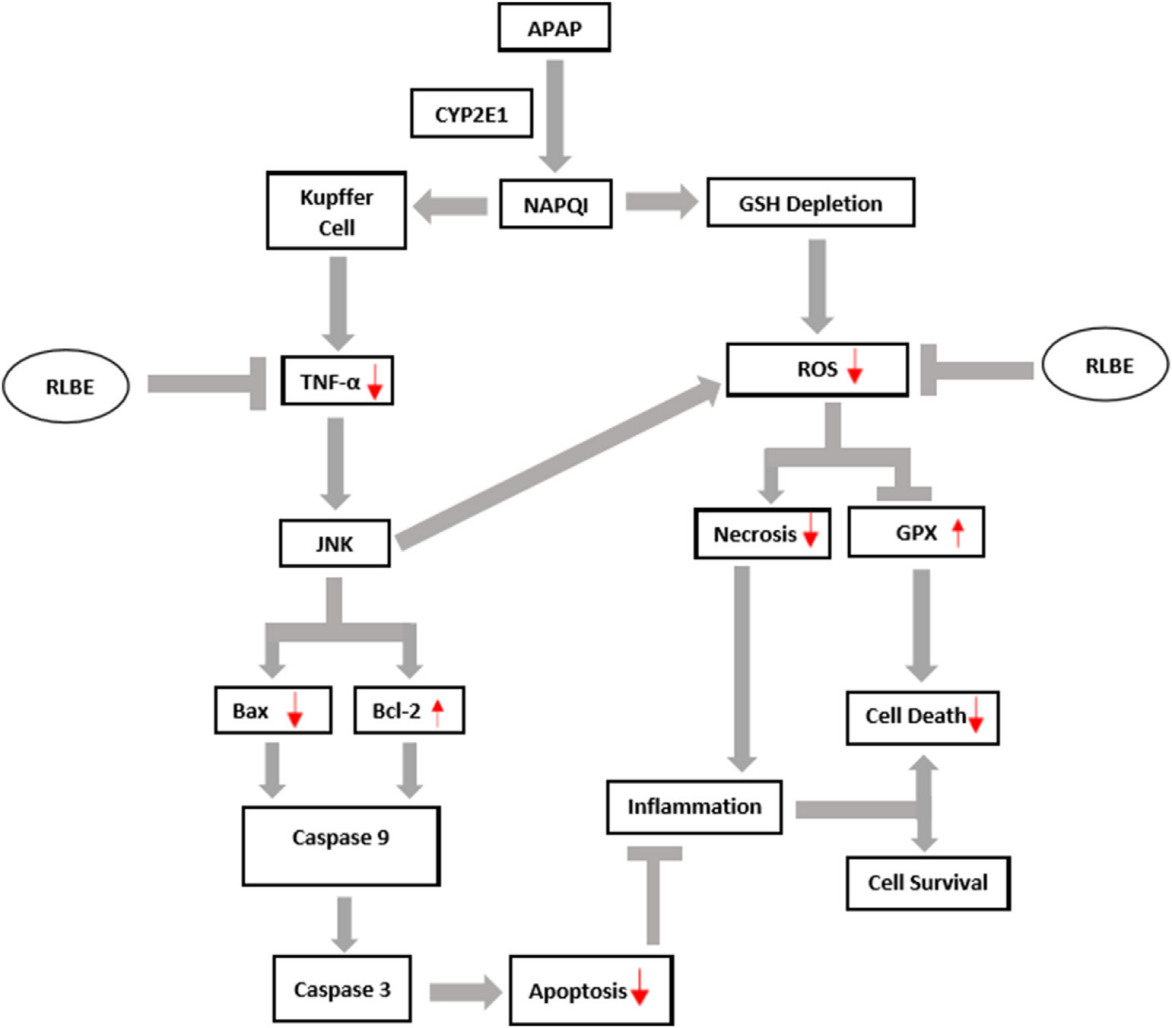
Proposed RLBE hepatoprotective mechanism in liver injury model. ∗CYP2E1 act to transform APAP to NAPQI. It induce GSH depletion then induce production of ROS. The excessive of ROS decrease the GPX gene expression leads to increase cell death. NAPQI activate the kupper cell and leads to TNF-α production that induce the JNK signaling pathways that also increase ROS level and leads to upregulate cell necrosis; increase inflammation; and induce cell death. While JNK induced the Bcl-2 down-regulation and Bax up-regulation, resulting in activation of caspase 9 and caspase 3 that leads to apoptosis cells. The RLBE treatments could inhibit the excessive ROS and TNF-α. It also could lowering the necrosis and apoptosis that leads to lowering inflammation. RBLE treatments decrease the cell death and increase the survival hepatic cells.

Based on this study, RBLE was shown have antioxidant, anti-necrotic, and anti-inflammatory activities. It mechanism as hepatoprotective agent in live injury was shown in Figure 6 that proposed by us based on the study result and literature review.

## 5 Conclusion

Red betel leaves extract treatments could reduce TNF-α level, reduce cell's apoptosis and increase live cells percentage, reduce intracellular ROS, reduce CYP2E1 and increase GPX level in HepG2 cells. This marked the hepatoprotective potential of RBLE through antioxidant, anti-necrotic, and anti-inflammatory activities. Further research on *in vivo* model is needed to confirm current result.

## Declarations

### Author contribution statement

C.N. Ginting, I.N.E. Lister and E. Girsang: Conceived and designed the experiments.

W. Widowati: Conceived and designed the experiments; Analyzed and interpreted the data.

D.T. Yusepany: Performed the experiments; Contributed reagents, materials, analysis tools or data.

A.M. Azizah: Analyzed and interpreted the data; Wrote the paper.

H.S.W. Kusuma: Contributed reagents, materials, analysis tools or data; Wrote the paper.

### Funding statement

This work was funded by Universitas Prima Indonesia, Medan, Indonesia.

### Declaration of interests statement

The authors declare no conflict of interest.

### Additional information

No additional information is available for this paper.
